# Decisional responsibility for mechanical ventilation and weaning: an international survey

**DOI:** 10.1186/cc10588

**Published:** 2011-12-14

**Authors:** Louise Rose, Bronagh Blackwood, Ingrid Egerod, Hege Selnes Haugdahl, José Hofhuis, Michael Isfort, Kalliopi Kydonaki, Maria Schubert, Riccardo Sperlinga, Peter Spronk, Sissel Storli, Daniel F McAuley, Marcus J Schultz

**Affiliations:** 1 Lawrence S. Bloomberg Faculty of Nursing, University of Toronto, 155 College St, Toronto, M5T 1P8, Canada; 2 Department of Respirology, Toronto East General Hospital, 825 Coxwell Ave, Toronto, M4C 3E7, Canada; 3 Department of Nursing, Mt Sinai Hospital, 600 University Ave, Toronto, M5G 1X5, Canada; 4 Li Ka Shing Institute, St Michael's Hospital, 30 Bond St, Toronto, M5B 1W8, Canada; 5 School of Nursing and Midwifery, Queen's University, 97 Lisburn Road, Belfast, BT9 7BL, Northern Ireland; 6 The University of Copenhagen, Faculty of Health Sciences and The University Hospitals Center for Nursing and Care Research, UCS, Copenhagen University Hospital Rigshospitalet, Department 7001, Blegdamsvej 9, DK-2100 Copenhagen O, Denmark; 7 Department for Research and Development, Levanger Hospital, Nord-Trøndelag Health Trust, Kirkegt. 2, Levanger, 7600, Norway; 8 Department of Intensive Care, Gelre Hospitals, Albert Schweitzerlaan 31, Apeldoorn, 7334DZ, The Netherlands; 9 Deutsches Institut für angewandte Pflegeforschung, Hülchrather Str. 15 Köln, 50670, Germany; 10 School of Health in Social Science Nursing Studies, The University of Edinburgh, 17/10 High Riggs, Edinburgh, EH3 9BW UK; 11 Faculty of Medicine, University of Basel, Institute of Nursing Science, Bernoullistr. 28, Basel 4056, Switzerland; 12 Center of Clinical Nursing Science, University Hospital Zurich, Raemistr. 100 (ZUR 44), Zurich 8091, Switzerland; 13 Faculty of Medicine and Surgery, Catholic University of the Sacred Heart, School of Nursing, Largo Francesco Vito, 1- 00168 Roma, Italy; 14 Cottolengo Hospital, Little House of Divine Providence, S.C. Training and Quality, School of Nursing, via Cottolengo 13, Turin, 10100, Italy; 15 Department of Intensive Care, Gelre Hospitals, Albert Schweitzerlaan 31, Apeldoorn, 7334DZ, The Netherlands; 16 Department of Health and Care Sciences, Faculty of Health Sciences, University of Tromsø, Romssa universitehta, N-9037, Norway; 17 Centre for Infection and Immunity, Room 01/017, Health Sciences Building, Queen's University, 97 Lisburn Road, Belfast BT9 7BL, Northern Ireland; 18 Department of Intensive Care Medicine, Academic Medical Center at the University of Amsterdam, Meibergdreef 9, Amsterdam, 1105 AZ, The Netherlands

## Abstract

**Introduction:**

Optimal management of mechanical ventilation and weaning requires dynamic and collaborative decision making to minimize complications and avoid delays in the transition to extubation. In the absence of collaboration, ventilation decision making may be fragmented, inconsistent, and delayed. Our objective was to describe the professional group with responsibility for key ventilation and weaning decisions and to examine organizational characteristics associated with nurse involvement.

**Methods:**

A multi-center, cross-sectional, self-administered survey was sent to nurse managers of adult intensive care units (ICUs) in Denmark, Germany, Greece, Italy, Norway, Switzerland, Netherlands and United Kingdom (UK). We summarized data as proportions (95% confidence intervals (CIs)) and calculated odds ratios (OR) to examine ICU organizational variables associated with collaborative decision making.

**Results:**

Response rates ranged from 39% (UK) to 92% (Switzerland), providing surveys from 586 ICUs. Interprofessional collaboration (nurses and physicians) was the most common approach to initial selection of ventilator settings (63% (95% CI 59 to 66)), determination of extubation readiness (71% (67 to 75)), weaning method (73% (69 to 76)), recognition of weaning failure (84% (81 to 87)) and weaning readiness (85% (82 to 87)), and titration of ventilator settings (88% (86 to 91)). A nurse-to-patient ratio other than 1:1 was associated with decreased interprofessional collaboration during titration of ventilator settings (OR 0.2, 95% CI 0.1 to 0.6), weaning method (0.4 (0.2 to 0.9)), determination of extubation readiness (0.5 (0.2 to 0.9)) and weaning failure (0.4 (0.1 to 1.0)). Use of a weaning protocol was associated with increased collaborative decision making for determining weaning (1.8 (1.0 to 3.3)) and extubation readiness (1.9 (1.2 to 3.0)), and weaning method (1.8 (1.1 to 3.0). Country of ICU location influenced the profile of responsibility for all decisions. Automated weaning modes were used in 55% of ICUs.

**Conclusions:**

Collaborative decision making for ventilation and weaning was employed in most ICUs in all countries although this was influenced by nurse-to-patient ratio, presence of a protocol, and varied across countries. Potential clinical implications of a lack of collaboration include delayed adaptation of ventilation to changing physiological parameters, and delayed recognition of weaning and extubation readiness resulting in unnecessary prolongation of ventilation.

## Introduction

Optimal management of mechanical ventilation and weaning requires dynamic and collaborative decision making to minimize complications and avoid delays in the transition to extubation. Effective collaboration requires open, extensive, and coordinated communication as well as shared team goals and will result in improved quality of care, patient safety and discharge outcomes [1-3]. In the absence of collaboration, ventilation decision making may be fragmented, inconsistent, and delayed [4].

Previous studies exploring interprofessional responsibility for ventilation and weaning decision making in Australia, New Zealand, and Denmark found that physicians and nurses actively collaborated in the management of ventilation and weaning, generally in the absence of protocols [5-8]. Nurses in Australia and New Zealand were frequently independently responsible for manipulation of ventilator settings titrated to physiologic parameters [9]. Intensive care unit (ICU) organizational characteristics have been noted as key contributors to ICU performance and patient outcomes [10]. Organizational characteristics such as staffing ratios, hierarchical structure, and ICU team functioning in the above countries may differ from those elsewhere [11].

International variation in aspects of the delivery of mechanical ventilation such as preferred ventilator mode, use of non-invasive ventilation (NIV), and adoption of protocols for weaning has been noted previously [12,13]. Interprofessional roles and responsibilities are influenced by differences in unit structure, staffing and skill-mix, patient case-mix, and medical and nursing leadership models [14]. The primary objective of our study was to describe the professional group with responsibility for determining key ventilation and weaning decisions including: selection of initial ventilator settings, titration of ventilator settings, weaning readiness, weaning method, extubation readiness, and weaning failure. We hypothesized that substantial variation would exist between and within countries for the professional group responsible for these decisions.

Our secondary objectives were to examine organizational characteristics associated with nurse involvement in ventilator and weaning decision making including nurse-to-patient ratios for invasive and non-invasive ventilation; perceived nursing autonomy and nursing contribution to ventilator decision-making; to describe the use of automated systems, protocols and/or guidelines for the management of mechanical ventilation and weaning; and the availability of education related to mechanical ventilation and associated processes for nurses. Results from the Netherlands have been previously reported in abstract form [15].

## Materials and methods

### Study Design and Sampling Frame

We conducted a multi-center, cross sectional, self-administered survey of adult ICUs in Denmark, Germany, Greece, Italy, Norway, Switzerland, the Netherlands and the United Kingdom (UK). These countries represent three of the four European sub-regions: Northern, Western, and Southern Europe. Our sample frame comprised all ICUs providing mechanical ventilation to critically ill adults in Denmark, Norway, Switzerland, the UK, and the Netherlands identified through existing intensive care networks. For example, ICUs in the UK were identified using the 2008 Directory of Critical Care [16]. Telephone contact was made to confirm that the ICU met inclusion criteria and to obtain nurse manager contact details. In Germany, Greece, and Italy we were unable to identify a reliable comprehensive list of all adult ICUs. Therefore nurse managers were recruited from existing personal e-mail lists within regions of a country (Attica in Greece and the Piedmont and Valle D'Aosta Regions in Italy) and nationally via advertisements in local journals and websites.

### Study Population

We included nurse managers of adult ICUs providing care to ventilated patients. We requested nurse managers consult senior medical colleagues and other senior nurses involved in the clinical management of ventilated patients to provide the best possible reflection of ventilation and weaning processes within their unit. If more than one nurse manager was employed in an ICU, we requested that only one survey be returned that reflected a consensus of opinion. Pediatric and neonatal ICUs or units not routinely providing mechanical ventilation such as coronary care and high dependency units were excluded.

### Survey Development and Testing

We administered a survey of mechanical ventilation and weaning responsibilities previously described and conducted in Australia and New Zealand [5]. Due to increased commercial availability of automated closed loop systems [17,18], we added a question on the use of SmartCare/PS™ (Dräger Medical, Lübeck, Germany), Adaptive Support Ventilation (ASV) (Hamilton Medical, Bonaduz, Switzerland), Proportional Assist Ventilation (PAV), and Mandatory Minute Ventilation (MMV). Questions relating to ICU demographic and staffing descriptions were contextually adapted for each country based on input from senior nurses and physicians. The survey (see Additional file 1) was then forward and back translated into the required languages. Country coordinators (fluent in English and the native language) and the principal investigator resolved inconsistencies in the two English versions (initial version and back-translated). Once the two English versions were consistent, the translated version was revised and checked by another native speaker. Prior to distribution, face validity was assessed in each country by a panel of experienced ICU nurses and physicians.

### Data Collection

Approval for conduct of the survey was obtained from ethics review boards and/or hospital administration in each country as required according to local guidelines. Return of a completed questionnaire was considered indicative of consent. Participants were advised that survey completion was voluntary. To maintain anonymity, no ICU or participant identifiers were collected.

A study investigator coordinated survey administration in each country. Surveys were distributed via mail in the UK and the Netherlands; by email in Denmark, Switzerland, Germany, Italy and Norway; and conducted by phone in Greece. In Germany a link to the survey was also advertised on professional websites. Country coordinators selected the survey delivery method based on available contact details. Three reminders to complete the survey were sent via mail, email or phone at two week intervals from initial distribution. Participants who received mail surveys were provided with a stamped-addressed envelope to return the survey to the coordinating center of that country. Email surveys were returned to the country coordinator (Denmark, Switzerland and Italy) or to a secure collector maintained by either Global Park http://www.globalpark.com/ (Germany) or Questback http://www.questback.com/ (Norway).

### Statistical Methods

We included data from incomplete questionnaires, therefore denominators for survey items vary. We excluded from analysis surveys with > 50% incomplete items. We summarized categorical variables such as professional group responsible for ventilation decisions using proportions and their 95% confidence intervals (CI). We calculated relative risks to determine the ventilator settings most likely to be adjusted by nurses > 50% of the time. The total scores for each of the two 0 to 10 scales used to measure perceived autonomy and nursing contribution to decision-making (0 represented no nurse autonomy and decision making input and 10 represented complete autonomy and nurse input into all decisions) were calculated and the median and interquartile range (IQR) determined.

Relevant variables selected *a priori *(country, nurse-to-patient ratio, presence of a protocol, hospital teaching status, number of ICU beds, and open versus closed model ICU) as likely to be associated with the professional group (collaborative compared to medical input only) most responsible for each of the six key decisions were examined using multiple logistic regression and odds ratios and their 95% CIs calculated. All models were assessed for collinearity and goodness of fit. All tests were two-tailed and we considered a *P*-value of 0.05 as statistically significant. Analyses were performed using SPSS 18.0 (SPSS, Chicago, IL, USA).

## Results

### Response Rates and Unit Characteristics

Response rates ranged from 39% (UK) to 92% (Switzerland) providing 586 surveys for evaluation. We were unable to determine the participant denominator in Germany due to the recruitment methods used and therefore cannot report a response rate. Unit characteristics are shown in Table 1. Germany had a higher proportion of ICUs with > 8 beds and more open ICUs than other countries except Denmark. A 1:1 nurse-to-patient ratio was used almost exclusively (93% of ICUs) for invasively ventilated patients in the UK; 6% used either a 1:1 or 1:2 nurse-to-patient ratio dependent on patient acuity. Switzerland, Denmark and Norway employed a 1:1 nurse-to-patient ratio in the majority of ICUs (61%, 73% and 90%, respectively). In the remaining countries, a 1:2 nurse-to-patient ratio was most common. For patients receiving NIV, a 1:2 nurse-to-patient ratio was most common in Germany, Switzerland, Italy, the Netherlands and the UK. ICUs in Denmark and Norway used a 1:1 nurse-to-patient ratio and Greek ICUs a 1:3 ratio.

**Table 1:** ICU Demographics

	Switzerland (*n *= 73)	UK (*n *= 115)	Germany (*n *= 201)	Netherlands (*n *= 71)	Denmark (*n *= 41)	Greece (*n *= 12)	Norway (*n *= 39)	Italy (*n *= 34)
Hospital type								
Private	6 (6)	-		-	-	-	-	-
University affiliated	11 (15)	71 (62)	64 (32)	3 (4)	22 (54)	5 (42)	11 (28)	1 (3)
Community/cantonal (teaching)	47 (64)	18 (16)	135 (67)	43 (61)	9 (22)	3 (25)	28 (72)	23 (68)
Community/cantonal (non-teaching)	9 (12)	21(18)	-	22 (31)	10 (24)	4 (33)	-	10 (29)
ICU specialty								
Med/surg/trauma	34 (47)	59 (51)	49 (24)	29 (41)	15 (37)	5 (42)	18 (46)	21 (62)
Medical/surgical	26 (36)	36 (31)	50 (25)	33 (47)	16 (39)	3 (25)	12 (31)	4 (12)
Medical (only)	5 (7)	-	38 (19)	-	2 (5)	-	2 (5)	2 (6)
Surgical (only)	3 (4)	3 (3)	24 (12)	2 (3)	-	-	3 (8)	2 (6)
Cardiothoracic	1 (1)	8 (7)	14 (7)	1 (1)	3 (7)	4 (33)	1 (3)	4 (12)
Neuro/trauma	3 (4)	4 (4)	15 (8)	2 (3)	2 (5)	-	1 (3)	-
Neurosurgery (only)	1 (1)	-	5 (3)	-	3 (7)	-	1 (3)	-
Burns	-	1 (1)	1 (1)	-	-	-	1 (3)	-
ICU type								
closed	66 (90)	97 (84)	142 (71)	44 (62)	9 (22)	7 (58)	30 (77)	32 (94)
open	6 (8)	12 (10)	56 (28)	15 (21)	22 (54)	3 (25)	8 (21)	1 (3)
ICU bed numbers								
≤8 beds	44 (60)	59 (51)	36 (18)	34 (48)	19 (46)	5 (42)	25 (64)	28 (82)
9-16 beds	18 (25)	31 (27)	116 (58)	26 (37)	11 (27)	6 (50)	7 (18)	6 (18)
≥17 beds	7 (10)	16 (21)	48 (24)	9 (13)	8 (20)	1 (8)	5 (13)	

All data are n (%). Percentages may not add up to 100% due to missing data or rounding. Med, medical; n, number; surg, surgical.

### Decisional Responsibility

Interprofessional responsibility for six key ventilation and weaning decisions is shown by country in Table 2. Interprofessional collaboration was the most common approach for all decisions regarding (n/N, % (95% CI)) initial selection of ventilator settings (365/584, 63% (59-66)), determination of extubation readiness (414/581, 71% (67-75)), weaning method (423/583, 73% (69-76)), recognition of weaning failure (489/582, 84% (81-87)) and weaning readiness (496/585, 85% (82-87)), and titration of ventilator settings (515/582, 88% (86-91)). Despite interprofessional collaboration being least likely in the selection of initial ventilator settings, nurses collaborated in this decision in > 75% of respondent ICUs in Switzerland, Germany and the UK.

**Table 2:** Responsibility for Ventilation Decisions by Country

	Switzerland (*n *= 73)	UK (*n *= 115)	Germany (*n *= 201)	Netherlands (*n *= 71)	Denmark (*n *= 41)	Greece (*n *= 12)	Norway (*n *= 39)	Italy (*n *= 34)
Initial ventilator settings								
Physician only	13 (18)	23 (20)	49 (24)	32 (45)	18 (44)	7 (58)	25 (64)	23 (68)
Collaborative	55 (85)	90 (78)	134 (67)	40 (56)	22 (54)	5 (42)	14 (36)	11 (32)
Nurse only	4 (6)	1 (1)	18 (9)	-	-	-	-	-
Other	1 (1)	-	-	1 (1)	-	-	-	-
Titration of ventilator settings								
Physician only	3 (4)	-	8 (4)	3 (4)	2 (5)	1 (8)	3 (8)	15 (44)
Collaborative	63 (86)	112 (99)	176 (88)	66 (93)	37 (90)	11 (92)	34 (87)	19 (56)
Nurse only	5 (6.8)	1 (1)	15 (8)	2 (3)	1 (2)	-	2 (5)	-
Other	2 (3)	1 (1)	-	-	-	-	-	-
Readiness to wean								
Physician only	5 (7)	4 (4)	25 (12)	11 (16)	1 (2)	8 (67)	7 (18)	18 (53)
Collaborative	65 (89)	111 (97)	172 (82)	59 (83)	39 (95)	4 (33)	32 (82)	16 (47)
Nurse only	2 (3)	-	3 (2)	-	-	-	-	-
Other	1 (1)	4 (4)	-	1 (1)	-	-	-	-
Weaning method								
Physician only	5 (7)	10 (9)	37 (18)	20 (28)	10 (24)	6 (50)	11 (28)	25 (74)
Collaborative	59 (81)	104 (90)	131 (71)	49 (69)	31 (76)	6 (50)	27 (69)	9 (27)
Nurse only	4 (6)	-	22 (11)	1 (1)	-	-	-	-
Other	5 (7)	6 (5)	-	1 (1)	-	-	-	-
Readiness to extubate								
Physician only	17 (23)	15 (13)	45 (22)	35 (49)	9 (22)	7 (58)	13 (33)	20 (59)
Collaborative	56 (77)	98 (85)	152 (76)	36 (49)	32 (78)	5 (42)	25 (64)	14 (41)
Nurse only	-	-	2 (1)	-	-	-	-	-
Other	-	2 (2)	-	-	-	-	-	-
Weaning failure								
Physician only	7 (10)	2 (2)	13 (7)	7 (10)	4 (10)	6 (50)	6 (15)	12 (35)
Collaborative	65 (89)	105 (91)	169 (85)	57 (80)	37 (90)	6 (50)	32 (82)	21 (62)
Nurse only	1 (1)	5 (4)	17 (9)	3 (4)	-	-	-	-
Other	-	2 (2)	-	4 (6)	-	-	-	-

All data are n (%). Note some % do not add to 100 due to missing data. Other: includes physiotherapists or specialized ventilator practitioners who may also hold a nursing qualification. n, number

A nurse-to-patient ratio other than 1:1 was associated with decreased interprofessional collaboration (decisions made independently by physicians without nursing input) during titration of ventilator settings (OR 0.2 (95% CI 0.1-0.6)), weaning method (0.4 (0.2-0.9)), determination of extubation readiness (0.5 (0.2-0.9)) and weaning failure (0.4 (0.1-1.0)) when controlling for country, ICU type (open versus closed), ICU size, presence of a protocol, and hospital teaching status. Use of a ventilator protocol or guideline was associated with increased collaborative decision making (communication between physicians and nurses) for weaning (OR 1.8 (95% CI 1.0-3.3)) and extubation readiness (1.9 (1.2-3.0)) and weaning method (1.8 (1.1-3.0)) when controlling for the same variables. Country of ICU location influenced the profile of professional responsibility for all decisions. Nurses were least likely to be involved in any type of ventilator decision making in ICUs located in Greece and Italy and most likely to be involved in Switzerland and the UK. Nurses were independently (without consulting a physician) responsible for titration of Fraction of inspired oxygen (FiO_2_) and pressure support in most ICUs (Table 3). In the majority of ICUs in Switzerland, Germany and the UK, nurses independently titrated all ventilator settings including change of mode except the level of positive end expiratory pressure (PEEP). Nurses rarely independently titrated ventilator settings in Italy and Greece.

**Table 3:** Type of Ventilator Settings made independently by Nurses*

	n/N	% (95% CI)	RR (95% CI)
Decrease of FiO_2_	392/580	68 (64-71)	1
Increase of FiO_2 _	386/580	67 (63-70)	1.0 (0.9-1.1)
Increase of pressure support	321/582	55 (51-59)	0.8 (0.7-0.9)
Decrease of pressure support	317/580	55 (51-59)	0.8 (0.7-0.9)
Titration of respiratory rate	290/581	50 (46-54)	0.7 (0.7 -0.8)
Titration of tidal volume	251/576	44 (40-48)	0.6 (0.6-0.7)
Titration of inspiratory pressure	229/577	40 (36-44)	0.6 (0.5-0.7)
Change mode	231/583	40 (36-44)	0.6 (0.5-0.7)
Decrease of PEEP	162/579	28 (25-32)	0.4 (0.4-0.5)
Increase of PEEP	147/581	25 (22-29)	0.4 (0.3-0.4)

*Nurse responsible to make and implement ventilator setting change independent > 50% of the time.

CI, confidence interval; RR, relative risk; FiO_2_, fraction of inspired oxygen; number; PEEP, positive end expiratory pressure.

### Automated Closed Loop Systems and Ventilation Protocols

**Figure 1:**
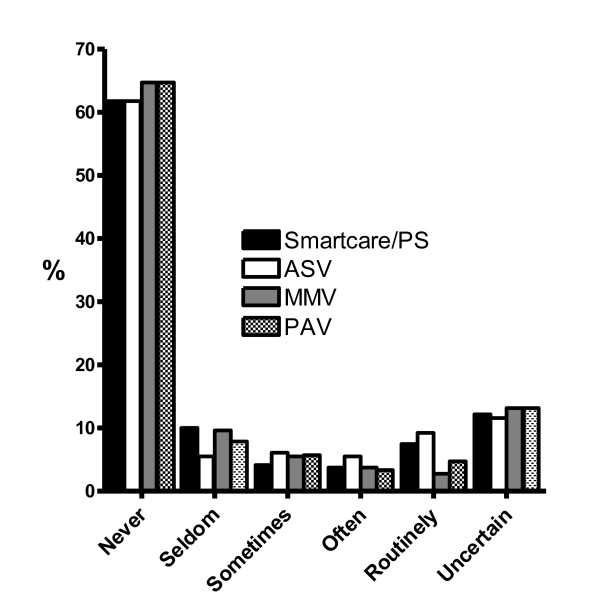
**Use of Automated Closed Loop Systems**.

Of the 586 ICUs, 319 (55% [50-59]) indicated they used SmartCare/PS, ASV, PAV or MMV. More ICUs reported using ASV > 50% of the time than other automated weaning systems (Figure 1). Protocols for ventilation (54% in the UK to 81% in Switzerland) and weaning (56% in Italy to 69% in Switzerland) were used in most ICUs in all countries with the exception of Greece where no ICU reported a protocol for management of ventilation and only one ICU reported availability of a weaning protocol. Availability of protocols for NIV ranged from 1 in 12, 8% (Greece) to 62 in 71, 87% (Netherlands) ICUs.

### Nurse Autonomy, Influence and Ventilator Education

Nurse managers' ratings of nurse autonomy and influence on decision making about mechanical ventilation practices ranged from 0 (no autonomy or influence) to 10 (complete autonomy and always influenced decisions) with a median score of 7 for both scales. Autonomy was rated highest by Swiss nurse managers (median 8, IQR 6 to 8) and lowest by those from Greek ICUs (5, 5 to 7). Ratings of nurse influence in ventilation decision making were similar across all countries. Most ICUs in all countries provided education related to ventilation to nurses during commencement of employment (65% in Italy [lowest] to 98% in the UK [highest]).

## Discussion

Our findings indicate, according to nurse managers, that interprofessional collaboration was the predominant model for decisions about mechanical ventilation and weaning and nurses generally had a reasonable influence on decisions made. Interprofessional collaboration varied according to the type of decision with physicians more likely to select initial ventilator settings and nurses more involved in the ongoing titration of ventilation and determination of extubation readiness. To the best of our knowledge, this is the largest survey describing interprofessional role responsibility for mechanical ventilation across Europe. Our findings suggest greater involvement of nurses in ventilator adjustment compared to a previous survey of physicians profiling ICU nursing in Western Europe conducted over a decade ago [14] but congruence in some countries with interprofessional responsibility for ventilation decisions in Australia and New Zealand [5] and participation in weaning practices reported in a pilot study of European nurses [19]. Physicians are generally present during initiation of ventilation as it coincides with intubation or arrival in ICU and represents an acute deterioration in respiratory status. ICU nurses maintain a near continuous presence at the bedside and therefore may be best positioned to titrate ventilator settings in response to changes in physiologic parameters.

Interprofessional collaboration for ventilation decision making varied by country with nursing input into ventilation decisions highest in Switzerland, Germany, and the UK and lowest in Greece and Italy. Irrespective of country, collaboration was influenced by nurse-to-patient ratio and the presence of protocols. Variation in the degree of collaboration is likely due to organizational, professional, and systemic factors that create power differences and delineate role responsibility [20]. The clinical implications of this variation in collaborative decision making on patient outcomes such as the duration of ventilation and weaning is unclear. However, several randomized controlled trials of either weaning or sedation protocols attribute the lack of reduction in ventilation duration to the existing organizational context including high staffing ratios, nurse autonomy in decision making and frequency of medical rounds that influence the usual care arm of the trial [21-23]. The absence of interprofessional collaboration has the potential to result in delayed adaptation of ventilation to changing physiological parameters, and delayed recognition of weaning and extubation readiness resulting in unnecessary prolongation of ventilation. High levels of interprofessional collaboration have previously been associated with low standardized mortality ratios (SMR) [3] and lower rates of ICU readmission following ICU discharge [24,25]; whereas ineffective interprofessional collaboration has been associated with the development of ventilator associated pneumonia and pressure ulcers [26], poor team functioning, morale [27] and ethical decision making [28]. Further studies are required to examine the impact of interprofessional collaboration on potentially modifiable outcomes such as weaning duration.

The utility of nurse involvement in ventilator decision making is reliant on appropriate knowledge and skills to manage ventilation. Differences existed in the proportion of ICUs providing education related to mechanical ventilation across countries, most probably as a reflection on the role of nurses in ventilation and weaning. On a broader level, differences also exist in the type and content of critical care nursing education internationally [29] that likely impact nurse involvement and autonomy. Furthermore, in ICUs with lower nurse-to-patient ratios, nurses may not be available for ventilator decision making due to the demands of ongoing assessment and provision of care to more than one patient.

Our findings suggest that nurses were more likely to make and implement decisions related to weaning, such as titration of pressure support and FiO_2_, independently. Nursing involvement in weaning has increased over the past two decades with the introduction of weaning protocols. Although, as discussed above, the effect of weaning protocols may be moderated by contextual factors [30] and no study has identified harm associated with nursing involvement in the weaning process. Interestingly, the level of PEEP was the ventilator setting least likely to be independently adjusted by nurses. While we did not seek information on the rationales for nurse initiated ventilator titration, we hypothesize that knowledge required to adjust PEEP may be viewed as more complex or that PEEP adjustment is perceived to pose greater risk of adverse consequences, although the logic of this viewpoint is questionable given the evidence of harm related to high tidal volume ventilation [31].

This study, to the best of our knowledge, is the first to report insight into the use of automated weaning systems in practice. Though just over 50% of ICUs reported using one or more automated closed loop system, few ICUs in all countries used them routinely. The goal of automated weaning systems is to improve adaptation of ventilatory support to the patients' needs through continuous monitoring and real-time interventions [17]. Several randomized controlled trials describe a reduction in duration of mechanical ventilation using automated weaning [32-34] while others demonstrate no effect [23,35]. As technology advances and the evidence about the utility of these systems increases, it is important to continue to track adoption into practice.

Our study has several limitations including the potential for selection bias, self-report bias, confounding, and lack of generalizability. Nurse managers interested in the delineation of interprofessional responsibilities for mechanical ventilation were probably more likely to respond to this questionnaire. As we chose to survey nurse managers, nurses' roles with respect to ventilation and weaning may be over-represented despite our instruction to confer with senior medical and nursing colleagues. There is also the possibility that other contextual factors not measured in our survey contributed to decisional responsibility. Lack of generalizability is particularly problematic regarding survey responses from Greece and Italy as only certain regions of these countries were surveyed.

## Conclusion

Our cross-sectional survey of nurse managers suggests that collaborative decision making for important decisions related to ventilation and weaning was employed in most ICUs in all countries. Nurse-to-patient ratio, availability of protocols and country influenced nurse involvement in decision making. Further study is warranted to determine if collaborative decision making is associated with improved patient outcomes for mechanically ventilated patients.

## Key messages

• interprofessional collaboration was the predominant model for decisions about mechanical ventilation and weaning

• interprofessional collaboration for ventilation decision making varied by country and was modified by nurse-to-patient ratio and the presence of protocols

• more than 50% of ICUs reported using one or more automated closed loop system, few ICUs in all countries used them routinely

• variation exists in the adoption of protocols for ventilation and weaning

## Abbreviations

ASV: adaptive support ventilation; CI: confidence interval; ICU: intensive care unit; IQ: interquartile range; MMV: mandatory minute ventilation; NIV non invasive ventilation; OR: odds ratio; PAV: proportional assist ventilation; PEEP: positive end expiratory pressure.

## Competing interests

The authors declare that they have no competing interests.

## Authors' contributions

LR, MJS, PS, IE, BB, HH conceived the study. BB, DM, IE, HH, JH, MI, KK, MS, RS coordinated data collection and data entry in their own countries. LR collated and analyzed the data. All authors contributed to manuscript drafts and have read and approved the final manuscript.

## Supplementary Material

Additional file 1 **Survey of Ventilation and Weaning Responsibility**. Generic version of the survey in English. Click here for file
