# Supplementary material for: Decisional responsibility for mechanical ventilation and weaning: an international survey
Source: Crit Care. 2011 Dec 14;15(6):R295. doi: 10.1186/cc10588 (PMC3388643; doi:10.1186/cc10588)
Supplement: Additional file 1 — Survey of Ventilation and Weaning Responsibility. Generic version of the survey in English. [file cc10588-S1.DOC]

| **Survey of Mechanical Ventilation and Weaning Role Responsibilities** | |
| --- | --- |
| **1. Who determines the initial selection of ventilator settings?** | |
| 🔿 Physicians only | 🔿 Nurses only |
| 🔿 Physicians and nurses in collaboration | 🔿 Other, please specify _______________ |
| **a. Identify the seniority of physicians responsible for initial selection of ventilator settings** | |
| 🔿 Consultants only | 🔿 Registrars and above |
| 🔿 Residents and above | 🔿 Other, please specify _______________ |
| **b. If applicable, identify the seniority of nurses responsible for initial selection of ventilator settings** | |
| 🔿 Senior nurses only (e.g. clinical nurse specialists, nurse managers, educators) | |
| 🔿 All nursing staff (once oriented to the ICU environment) | |
| 🔿 Other, please specify _____________________________________________ | |
| **2. Who evaluates the patient’s response to mechanical ventilation and titrates settings if required?** | |
| 🔿 Physicians only | 🔿 Nurses only |
| 🔿 Physicians and nurses in collaboration | 🔿 Other, please specify _______________ |
| **a. Identify the seniority of physicians responsible for titration of ventilator settings** | |
| 🔿 Consultants only | 🔿 Registrars and above |
| 🔿 Residents and above | 🔿 Other, please specify _______________ |
| **b. If applicable, identify the seniority of nurses responsible for titration of ventilator settings** | |
| 🔿 Senior nurses only (e.g. clinical nurse specialists, nurse managers, educators) | |
| 🔿 All nursing staff (once oriented to the ICU environment) | |
| 🔿 Other, please specify _____________________________________________ | |
| **3. Who decides when a patient is ready to wean?** | |
| 🔿 Physicians only | 🔿 Nurses only |
| 🔿 Physicians and nurses in collaboration | 🔿 Other, please specify _______________ |
| **a. Identify the seniority of physicians responsible for determining weaning readiness** | |
| 🔿 Consultants only | 🔿 Registrars and above |
| 🔿 Residents and above | 🔿 Other, please specify _______________ |
| **b. If applicable, identify the seniority of nurses responsible for determining weaning readiness** | |
| 🔿 Senior nurses only (e.g. clinical nurse specialists, nurse managers, educators) | |
| 🔿 All nursing staff (once oriented to the ICU environment) | |
| 🔿 Other, please specify _____________________________________________ | |
| **4. Who decides the method of weaning from mechanical ventilation?** | |
| 🔿 Physicians only | 🔿 Nurses only |
| 🔿 Physicians and nurses in collaboration | 🔿 Other, please specify _______________ |

| **a. Identify the seniority of physicians responsible for determining the method of weaning** | |
| --- | --- |
| 🔿 Consultants only | 🔿 Registrars and above |
| 🔿 Residents and above | 🔿 Other, please specify _______________ |
| **b. If applicable, identify the seniority of nurses responsible for determining the method of weaning** | |
| 🔿 Senior nurses only (e.g. clinical nurse specialists, nurse managers, educators) | |
| 🔿 All nursing staff (once oriented to the ICU environment) | |
| 🔿 Other, please specify _____________________________________________ | |
| **5. Who decides when a patient is ready to extubate?** | |
| 🔿 Physicians only | 🔿 Nurses only |
| 🔿 Physicians and nurses in collaboration | 🔿 Other, please specify _______________ |
| **a. Identify the seniority of physicians responsible for determining readiness for extubation** | |
| 🔿 Consultants only | 🔿 Registrars and above |
| 🔿 Residents and above | 🔿 Other, please specify _______________ |
| **b. If applicable, identify the seniority of nurses responsible for determining readiness for extubation** | |
| 🔿 Senior nurses only (e.g. clinical nurse specialists, nurse managers, educators) | |
| 🔿 All nursing staff (once oriented to the ICU environment) | |
| 🔿 Other, please specify _____________________________________________ | |
| **6. Who decides when a patient is failing a weaning trial?** | |
| 🔿 Physicians only | 🔿 Nurses only |
| 🔿 Physicians and nurses in collaboration | 🔿 Other, please specify _______________ |
| **a. Identify the seniority of physicians responsible for determining weaning failure** | |
| 🔿 Consultants only | 🔿 Registrars and above |
| 🔿 Residents and above | 🔿 Other, please specify _______________ |
| **b. If applicable, identify the seniority of nurses responsible for determining weaning failure** | |
| 🔿 Senior nurses only (e.g. clinical nurse specialists, nurse managers, educators) | |
| 🔿 All nursing staff (once oriented to the ICU environment) | |
| 🔿 Other, please specify _____________________________________________ | |
| **7. What is the nurse-to-patient ratio for patients receiving mechanical ventilation in your ICU?** | |
| 🔿 1:1 ratio | 🔿 1:2 ratio |
| 🔿 1:3 ratio | 🔿 Other, please specify: ______________ |
| **8. What is the nurse-to-patient ratio for patients receiving non-invasive ventilation in your ICU?** | |
| 🔿 1:1 ratio | 🔿 1:2 ratio |
| 🔿 1:3 ratio | 🔿 Other, please specify: ______________ |

| **9. How would you rate nursing autonomy in regards to mechanical ventilation practices? Please circle the number on the scale below** | | | | | | | | | | | | | | | | | | | | | | | | | |
| --- | --- | --- | --- | --- | --- | --- | --- | --- | --- | --- | --- | --- | --- | --- | --- | --- | --- | --- | --- | --- | --- | --- | --- | --- | --- |
| 0  **No autonomy** | 1 | 2 | 3 | | | 4 | | | 5 | | 6 | | | 7 | | | | 8 | | | 9 | | 10  **Complete autonomy** | | |
| Please comment (optional)  _________________________________________________________________________________  _________________________________________________________________________________  _________________________________________________________________________________  _________________________________________________________________________________  _________________________________________________________________________________ | | | | | | | | | | | | | | | | | | | | | | | | | |
| **10. How often do nursing contributions influence decisions made regarding mechanical ventilation? Please circle the number on the scale below** | | | | | | | | | | | | | | | | | | | | | | | | | |
| 0  **Never** | 1 | 2 | 3 | | | 4 | | | 5 | | 6 | | | 7 | | | | 8 | | | 9 | | 10  **Always** | | |
| Please comment (optional)  _________________________________________________________________________________  _________________________________________________________________________________  _________________________________________________________________________________  _________________________________________________________________________________  _________________________________________________________________________________ | | | | | | | | | | | | | | | | | | | | | | | | | |
| **11. How often do nurses *make* and *implement* the following decisions *independently* (without prior direct consultation with a physician):** | | | | | | | | | | | | | | | | | | | | | | | | | |
|  | | | | | **Never** (0%) | | | **Seldom** (1-25%) | | | | Frequently (26-50%) | | | | **Often** (51-75%) | | | | **Routinely** (>75%) | | | **Uncertain** | | |
| Change of mode | | | | | 🔿 | | | 🔿 | | | | 🔿 | | | | 🔿 | | | | 🔿 | | | 🔿 | | |
| Titration of respiratory rate | | | | | 🔿 | | | 🔿 | | | | 🔿 | | | | 🔿 | | | | 🔿 | | | 🔿 | | |
| Titration of tidal volume | | | | | 🔿 | | | 🔿 | | | | 🔿 | | | | 🔿 | | | | 🔿 | | | 🔿 | | |
| Titration of inspiratory pressure | | | | | 🔿 | | | 🔿 | | | | 🔿 | | | | 🔿 | | | | 🔿 | | | 🔿 | | |
| Increase of pressure support | | | | | 🔿 | | | 🔿 | | | | 🔿 | | | | 🔿 | | | | 🔿 | | | 🔿 | | |
| Decrease of pressure support | | | | | 🔿 | | | 🔿 | | | | 🔿 | | | | 🔿 | | | | 🔿 | | | 🔿 | | |
| Increase of PEEP | | | | | 🔿 | | | 🔿 | | | | 🔿 | | | | 🔿 | | | | 🔿 | | | 🔿 | | |
| Decrease of PEEP | | | | | 🔿 | | | 🔿 | | | | 🔿 | | | | 🔿 | | | | 🔿 | | | 🔿 | | |
| Increase of FiO_2_ | | | | | 🔿 | | | 🔿 | | | | 🔿 | | | | 🔿 | | | | 🔿 | | | 🔿 | | |
| Decrease of FiO_2_ | | | | | 🔿 | | | 🔿 | | | | 🔿 | | | | 🔿 | | | | 🔿 | | | 🔿 | | |
| **12. In your ICU, do you have a guideline/policy/protocol for management of mechanical ventilation?** | | | | | | | | | | | | | | | | | | | | | | | | | |
| 🔿 Yes | | | | 🔿 No | | | | | | | | | | | 🔿 Uncertain | | | | | | | | | | |
| **13. In your ICU, do you have a guideline/policy/protocol for weaning from mechanical ventilation?** | | | | | | | | | | | | | | | | | | | | | | | | | |
| 🔿 Yes | | | | 🔿 No | | | | | | | | | | | 🔿 Uncertain | | | | | | | | | | |
| **If Yes, does it contain information on management of patients failing weaning?** | | | | | | | | | | | | | | | | | | | | | | | | | |
| 🔿 Yes | | | | 🔿 No | | | | | | | | | | | 🔿 Uncertain | | | | | | | | | | |
| **14. In your ICU, do you have a guideline/policy/protocol for management of non-invasive ventilation?** | | | | | | | | | | | | | | | | | | | | | | | | | |
| 🔿 Yes | | | | 🔿 No | | | | | | | | | | | 🔿 Uncertain | | | | | | | | | | |
| 1**5. Are any of the following automated weaning modes used in your ICU?** | | | | | | | | | | | | | | | | | | | | | | | | | |
|  | | | | | | | **Never** (0%) | | | **Seldom** (1-25%) | | | Frequently  (26-50%) | | | | **Often** (51-75%) | | | | | **Routinely** (>75%) | | | **Uncertain** |
| SmartCare/PS™ | | | | | | | 🔿 | | | 🔿 | | | 🔿 | | | | 🔿 | | | | | 🔿 | | | 🔿 |
| Adaptive support ventilation (ASV) | | | | | | | 🔿 | | | 🔿 | | | 🔿 | | | | 🔿 | | | | | 🔿 | | | 🔿 |
| Mandatory minute ventilation (MMV) | | | | | | | 🔿 | | | 🔿 | | | 🔿 | | | | 🔿 | | | | | 🔿 | | | 🔿 |
| Proportional assist ventilation (PAV) | | | | | | | 🔿 | | | 🔿 | | | 🔿 | | | | 🔿 | | | | | 🔿 | | | 🔿 |
| **NURSE EDUCATION** | | | | | | | | | | | | | | | | | | | | | | | | | |
| **1. Do nurses receive education on ventilation during ICU orientation?** | | | | | | | | | | | | | | | | | | | 🔿 Yes | | | 🔿 No | | 🔿 Uncertain | |
| If YES, please describe (optional)  ____________________________________________________________________________________________________  ____________________________________________________________________________________________________  ____________________________________________________________________________________________________ | | | | | | | | | | | | | | | | | | | | | | | | | |
| **2. Are opportunities available in your ICU for ongoing professional development related to mechanical ventilation?** | | | | | | | | | | | | | | | | | | | 🔿 Yes | | | 🔿 No | | 🔿 Uncertain | |
| If YES, please describe (optional)  ____________________________________________________________________________________________________  ____________________________________________________________________________________________________  ____________________________________________________________________________________________________ | | | | | | | | | | | | | | | | | | | | | | | | | |
| | **3. Number of nurses holding a post-graduate critical care specialty qualification:** _________ | | | | | | | | | | | | | | | --- | --- | --- | --- | --- | --- | --- | --- | --- | --- | --- | --- | --- | --- | | **ICU DEMOGRAPHICS** | | | | | | | | | | | | | | | **1.Hospital type** | | 🔿 University affiliated | | | | 🔿 Community/teaching | | | | | | 🔿 Community/non-teaching | | | **2. Please identify the primary specialty of your ICU** | | | | | | | | | | | | | | | 🔿 Surgical (only) | | | 🔿 Cardiovascular (only) | | | | 🔿 Trauma/ Neuro | | | | 🔿 Mixed medical/surgical | | | | 🔿 Medical (only) | | | 🔿 Neuroscience (only) | | | | 🔿 Burns (only) | | | | 🔿 Mixed medical/surgical/trauma | | | | **3. ICU type** | 🔿 Closed (intensivist-led) | | | | 🔿 Open (under the care of physicians from any specialty) | | | | | | | | | | **4. Number of ICUs in the hospital** | | | | _____________ | | | | | | **Number of ICU beds** ______________ | | | | | **5. Please identify the approximate number of beds staffed in your hospital** | | | | | | | | | | | | | | | 🔿 <100 | | | | | | | | 🔿 401 - 750 | | | | | | | 🔿 100 - 400 | | | | | | | | 🔿 >750 | | | | | | | **6. Please identify the actual number as well as the full-time equivalents of the following staff members:** | | | | | | | | | | | | | | | Number of Registered Nurses (RNs) (total) | | | | |  | | | | Full time equivalent (FTE) of RNs (total) | | | |  | | Number of Registered Practical Nurses | | | | |  | | | | FTE of RPNs | | | |  | | Number of Clinical Nurse Specialists (CNSs) | | | | |  | | | | FTE of CNSs | | | |  | | Number of Nurse Educators | | | | |  | | | | FTE of Nurse Educators | | | |  | | Number of Medical Consultants | | | | |  | | | | FTE of Medical Consultants | | | |  | | Number of Registrars | | | | |  | | | | FTE of Registrars | | | |  | | Number of Residents | | | | |  | | | | FTE of Residents | | | |  | | | | | | | | | | | | | | | | | | | | | | | | | | |
